# Synergistic effect of endurance training and nettle leaf extract on the IDO1-KYN-AHR pathway homeostasis and inhibiting of liver toxicity in rats with STZ-induced diabetes

**DOI:** 10.3389/fendo.2023.1071424

**Published:** 2023-05-26

**Authors:** Rouhollah Haghshenas, Younes Aftabi, Saied Doaei, Maryam Gholamalizadeh

**Affiliations:** ^1^ Department of Sport Sciences, Faculty of Humanities, Semnan University, Semnan, Iran; ^2^ Tuberculosis and Lung Diseases Research Center, Tabriz University of Medical Sciences, Tabriz, Iran; ^3^ Department of Community Nutrition, School of Nutrition and Food Sciences, Shahid Beheshti University of Medical Sciences, Tehran, Iran; ^4^ Cancer Research Center, Shahid Beheshti University of Medical Sciences, Tehran, Iran

**Keywords:** AHR pathway, kynurenine, IDO1, diabetes, exercise, *Urtica dioica* L

## Abstract

**Introduction:**

Diabetes adversely affects a number of hepatic molecular pathways, including the kynurenine (KYN) pathway. KYN is produced by indoleamine 2,3-dioxygenase (IDO) and activates the aryl hydrocarbon receptor (AHR). This study evaluated the effect of endurance training (EndTr) and nettle leaf extract (NLE) on the IDO1-KYN-AHR pathway in the livers of rats with streptozotocin-induced diabetes.

**Methods:**

We divided 48 rats into six groups: controls (Ct), treated with EndTr (EndTr), diabetes-induced (D), D treated with NLE (D + NLE), D treated with EndTr (D + EnTr), and D treated with EndTr and NLE (D + EndTr + NLE). EndTr, D + EnTr, and D + EndTr + NLE groups were subjected to training with running on treadmill for 8 weeks, 5 days per week, 25 min in first session to 59 min at last session with intensity of 55% to 65% VO2max. Using real-time PCR gene (*Ahr*, *Cyp1a1*, and *Ido1*) expressions and ELISA, malondialdehyde (MDA) and protein (IDO1, AHR, and CYP1A1) levels were determined in the liver samples.

**Results:**

A significant three-way interaction of exercise, nettle, and diabetes was observed on the all variables (P< 0.001). In particular, significant increases in blood glucose level (BGL), in gene and protein expression, and in MDA and KYN levels were observed in the liver samples of the D group versus the Ct group (P< 0.05). BGL and liver MDA levels were significantly lower in the D + EndTr and D + NLE groups than that in the D group. However, the D + EndTr + NLE group showed a more significant decrease in these factors (P< 0.05). In addition, liver KYN levels were significantly lower in the EndTr group compared with that in the Ct group as well as in the D + EndTr + NLE and D + EndTr groups compared with that in the D groups (P< 0.05). Whereas both the EndTr and D + NLE groups showed lower *Ahr* expression and AHR level compared with the Ct and D groups, respectively (P< 0.05), the D + EndTr + NLE group showed a higher significant reduction in the AHR level than the D group (P< 0.05). The *Cyp1a1* expression and IDO1 level significantly decreased only in the D + EndTr + NLE group compared to that in the D group (P< 0.05).

**Conclusion:**

Overall, this study showed that the combination of EndTr and NLE may synergistically restore the imbalanced IDO1-KYN-AHR pathway in diabetic liver.

## 1 Introduction

Type 1 diabetes mellitus (T1D) is an autoimmune disease associated with the eradication of insulin-producing pancreatic β cells, chronic hyperglycemia, and many other pathological changes (1). Among them, as a result of the alteration of molecular mechanisms of liver physiology, hepatopathy is a prevalent pathological condition in diabetes (2, 3). Studies using T1D models have demonstrated that the kynurenine (KYN) pathway (KP) is one of the molecular pathways that is affected in the liver in this type of diabetes (4, 5). KYN is a derivative of tryptophan (TRP) that functions as an endogenous agonist for the aryl hydrocarbon receptor (AHR) and regulates a variety of immune and physiological functions (6). AHR, in turn, is a cytoplasm-resident ligand-activated transcription factor with many functions in health and disease, including diabetes and liver diseases (7–9). TRP 2,3-dioxygenase (TDO) and indoleamine 2,3-dioxygenase 1 (IDO1) and 2 (IDO2), whose expression is mediated by AHR signaling, catalyze the breakdown of TRP into KYN (10). Among these enzymes, IDO1 serves as an immunomodulator and plays a significant role in diabetes and liver disease (4, 11). Consequently, the IDO1-KYN-AHR pathway may have the potential to exert a profound influence on the pathophysiology of liver and diabetes.

Through kynurenic acid generation and/or increased levels of quinolinic acid (QA), KYN contributes to the pathophysiology of type 2 diabetes (T2D) (12, 13). Despite this, research on the changes in KP in T1D is limited, and little is known about perturbations in KP metabolism in this disorder (4). According to recent research, KP plays an imperative role in the severely dysregulated metabolic and immunologic milieu associated with ketoacidosis T1D (14). In addition, it has been demonstrated that KYN levels increase in the urine of patients with T1D, which may be related to systemic inflammation (15). Furthermore, some studies have shown that streptozotocin (STZ)–induced T1D increases QA and KYN production in the liver of rats (4, 5).

It has also been reported that both animal models and children with type 1 diabetes exhibit impaired expression and altered activity of IDO (16, 17). There is also evidence that elevated levels of IDO and QA contribute to the development of retinopathy in patients with T1D (18). There has also been speculation that decreased or absent expression of IDO1 in pancreatic β cells may be associated with T1D (19). Currently, it is assumed that restoration of IDO function is capable of restoring normoglycemia in T1D, and it might represent a useful target for the prevention/treatment of that disease (17, 20). As regards the involvement of components of the AHR pathway in T1D, Nguyen et al. reported a significant reduction in the expression of AHR nuclear translocator mRNA in multinucleated variant endothelial cells isolated from the heart of mice model of T1D (21). Furthermore, Yue et al. found that AHR modulates the function of immune cells, suppresses inflammatory cytokine production, and attenuates autoimmune reactions during T1D development (22). Recently, the IDO1-KYN-AHR pathway has been recognized as a promising therapeutic target for a variety of diseases, including cancer and diabetes (23, 24). Furthermore, it has been demonstrated in the literature that the KP can be used in the development of useful tools for prevent or treat T1D (25).

Currently, anti-diabetic therapy relies on conventional drugs, which are constrained in their efficacy. As such, medicinal herbs are increasingly being studied as complementary or alternative medicines in the treatment of diabetes (26, 27). The herb Nettle (*Urtica dioica* L.) has been widely used in traditional medicine to treat a number of ailments, including diabetes (28). Nettle leaf extract (NLE) reduces lipid peroxidation, increases antioxidant activity in the liver, and enhances glucose (Glc) homeostasis (29, 30). In mice with diabetes, it reduces blood Glc levels (BGLs) and attenuates cholestatic liver injury and fibrosis (31, 32). It has also been shown that NLE decreases liver damage by decreasing the inflammatory effects of STZ-induced diabetes in rats (33).

In addition to the use of pharmaceuticals, physical activity such as endurance training (EndTr) can also be used to manage and prevent diabetes (34, 35). EndTr has been demonstrated to have beneficial effects on diabetes-associated metabolic factors and contributes to liver health by inducing Glc-cognate pathways and inhibiting inflammation (36–38). Studies have demonstrated an interaction between physical exercise and the KP through the upregulation of KYN aminotransferase enzymes and the decrease of blood levels of KYN (39). Furthermore, exercise-induced KP may contribute to the prevention and treatment of chronic diseases through the modulation of energy homeostasis (40).

In addition, combining EndTr and NLE treatment has been tested in rats with STZ-induced diabetes, and the results have shown improved functions (41). However, no studies have been conducted on the effects of EndTr or NLE on IDO1-KYN-AHR pathway homeostasis in the liver under diabetic conditions. This study aimed to assess the effects of EndTr, NLE, and their combination on the IDO1-KYN-AHR pathway in the liver of rats with STZ-induced diabetes while taking into account the increasing incidence of T1D worldwide (42).

## 2 Methods

### 2.1 Experimental animals

A total of 48 male Wistar rats [age, 8–12 weeks; body weight (BW), 180 to 220 g] were purchased from the Pasteur Institute of Iran. In a polycarbonate cage, rats were subjected to a 12-h cycle of light and darkness. The humidity level was 65% ± 5%, and the temperature was adjusted to 25°C. Food (rat chow) and water were provided on *ad libitum* basis. The Regional Research Ethics Committee of Semnan University of Medical Sciences and Health Services approved all experimental protocols (IR.SEMUMS.REC.1399.291). A guide for the care and uses of laboratory animals published by the US National Institutes of Health was followed during the conduct of the study.

### 2.2 Preparation of nettle leaf extract and phytochemical analysis

Hydro-alcoholic extracts of nettle leaf were prepared according to previous reports (43, 44). Dried leaves of nettle were purchased from an herbal medicine shop. The extraction method was cold maceration with an ethanol solvent (70%). Briefly, leaves were finely ground, and 100 g of the produced powder was macerated in 1,000 ml of 70% ethanol (herb/solvent ratio of 1:10 w/v) for 72 h with intermittent stirring. After filtering, the solvent was evaporated in a rotary evaporator at 50°C. The powder was kept at −20°C until use. The powder was dissolved in distilled water at the time of administration. The herb/extract ratio was 8:1 (w/w). Phytochemical analysis of the hydro-alcoholic extract of nettle leaf was done using the gas chromatography method by a model HP-6890N Gas chromatography–mass spectrometry (GC-MS) coupled to a 5975 mass spectrometer (Agilent Technologies, Palo Alto, CA, USA) combined with a TDU (Thermal Desorption Unit) and cooling injector system (CIS4, Gerstel). Chromatographic separation was done in an HP5MS-UI column (Agilent Technologies) with helium as the gas carrier in constant pressure mode and split ratio 1:50. Initial temperature was 50°C, increasing at a ratio of 5°C min^−1^ until 70°C held for 1 min. In the next step, the temperature was increased to 240°C at 10°C min^−1^ and held for 15 min.

The mass spectrometer operated at 70-eV ionization voltage. Source and quadrupole temperatures were 230°C and 150°C, respectively. The mass range was 30.0 to 450.0 uma at 4 scan/s. Mass Selective Detector (MSD) transfer line was maintained at 280°C. We used ChemStation software (version E.02.02 SP1, Agilent Technologies) to acquire chromatograms. Compounds were qualitatively identified by comparison using the Wiley10th-NIST11b mass spectral database (Agilent Technologies, Wilmington, DE, USA).

### 2.3 *In silico* target analysis and gene enrichment

PubChem (https://pubchem.ncbi.nlm.nih.gov/) was searched to find SMILES notations of the detected compounds. As an alternative to PubChem, we used the OPSIN tool (https://opsin.ch.cam.ac.uk/), which provides an algorithm for interpreting most organic chemical nomenclature (45) to generate the appropriate SMILES notations for those chemicals without information available in PubChem. To perform further *in silico* target analysis, the SMILES notations were uploaded to the following servers. Using SwissADME (http://www.swissadme.ch), a tool that assesses the physicochemical properties, ADME (Absorption, Distribution, Metabolism, and Excretion) parameters, pharmacokinetic properties, drug-like characteristics, and medicinal chemistry friendliness of small molecules, we determined the absorption level, water solubility, and bioavailability scores of the chemicals. ProTox-II (https://tox-new.charite.de/protox_II/), which combines molecular similarity, fragment propensities, most frequent features, and (fragment similarity-based CLUSTER cross-validation) machine learning, based on 33 models predicting various toxicity endpoints (46) was applied to predict the LD50 of the chemicals and their toxicity class. For the purpose of identifying the target genes of chemicals, we used the SuperPred 3.0 server (http://prediction.charite.de). Using a machine learning algorithm and Morgan fingerprints of 2048 bits in length, SuperPred determines genes that may be targets of small molecules (47). Each chemical was assigned a specific target gene, as well as a combined target gene list for all chemical compounds (**Supplementary File 1**). An analysis of 15 lists was conducted using the ToppFun enrichment tool of the ToppGene Suite (http://toppgene.cchmc.org/enrichment.jsp). The ToppFun program performs functional enrichment of a gene list based on a wide range of omics and ontological data (48). A ranked list of diseases associated with each target gene list has been developed. The rank of “hyperglycemia” was considered to be an indicator of the direct relatedness of the target genes to diabetes and thus the corresponding chemical.

### 2.4 Diabetes induction

After adaptation to the laboratory environment, the diabetic model was induced in rats by intraperitoneal injection of STZ (Sigma-Aldrich, St. Louis, MO, USA) at a dose of 50 mg/kg BW. The STZ was dissolved in 0.1 ml of sodium citrate buffer (pH 4.5). On day 3 after the STZ injection, BGL from the fasted rats was measured, and rats with levels >150 mg/dl were considered to have diabetes. STZ-induced animals with diabetes murine models develop type 1 diabetes, due to the cytotoxic Glc analog STZ that is toxic to pancreatic β cells and causes insulin deficiency (49).

### 2.5 Animal treatment and training courses

Following the induction of diabetes, rats underwent a 2-week adaptation period. In addition, rats were divided into six groups: (1) Ct (control animals, n =8), (2) EndTr (rats treated with EndTr, n = 8), (3) D (rats with STZ-induced diabetes, n = 8), (4) D + NLE (rats with STZ-induced diabetes and treated with nettle leaves extract, n = 8), (5) D + EndTr (rats with STZ-induced diabetes and treated with EndTr, n = 8), and (6) D + EndTr + NLE (rats with STZ-induced diabetes and treated with EndTr and NLE, n = 8). Because of the numerous previous publications indicating that nettle consumption has hepatoprotective and antioxidative effects in the liver, as well as the beneficial effects of combining NLE and EndTr in healthy subjects (50–56), we did not include separate groups for investigating the NLE effects individually and in combination with EndTr in rats without diabetes. Animals in the exercise groups performed the designed protocol of training for 8 weeks (**Table 1A**) as described previously (59). The rats in those groups that were supposed to receive the extract were administered one dose per day of NLE (150 mg/kg BW) intragastrically 5 days a week for 8 weeks in accordance with previous reports (60). To get acquainted with the training protocol, animals in the exercise groups were trained on the treadmill by walking slowly for 5 min at a speed of 5 to 10 m/min. Subsequently, the rats in the exercise groups performed the EndTr protocol for 8 weeks and 5 days per week at 8–10 a.m. The indirect protocol was used to measure the aerobic capacity of rats. Training intensity was determined by VO2max and its relationship to treadmill speed and incline (61). After 2 weeks of initial familiarization with the treadmill, the aerobic capacity of the rats was assessed. Upon completing the initial warm-up, the test was initiated at a speed of 6 m/min. The treadmill speed was increased every 2 min by 1.8–2 m/min until the rats reached exhaustion. A protocol of training was designed on the basis of the obtained average of test speed and time. The intensity of training increased gradually during the training period.

**Table 1:** Training program and real-time PCR primers.

(A) Protocol of exercise training																							
Week	Time Intensity m/min																						Total time (Min)
	Time	3	3		3	3	3	3	3	3	3	3	3	3	3		3	3	3		3	Cool down 3 min 3m/min	
1	Warm-up for 5 min with the intensity of 10 m/min	10*	10		12	14	16																23
		
2		10	12		14	16	16	16	18														29
		
3		10	12		14	16	18	18	18	20													35
		
4		10	12		14	16	18	20	20	20	20	20	22										41
		
5		10	12		14	16	18	20	22	22	22	22	22	22	24								47
		
6		10	12		14	16	18	20	22	24	24	24	24	24	24		24	26					53
		
7		10	12		14	16	18	20	22	24	26	26	26	26	26		26	26	26		28		59
		
8		10	12		14	16	18	20	22	24	26	26	26	26	26		26	26	26		28		59
(B) Primers																							
Gene accession no.				Sequences									Amplicon size (bp)			Annealing Tm (°C)				Ref.			
*Ahr*				Fw: 5′-TCACTGCGCAGAATCCCACATCC-3′									186			60				(57)			
NM_013149				Rv: 5′-TCGCGTCCTTCTTCATCCGTTAGC-3′																			
*Cyp1a1*				Fw: 5′-GTCCCGGATGTGGCCCTTCTCAAA-3′									109 bp			56							
NM_012540				Rv: 5′-TAACTCTTCCCTGGATGCCTTCAA-3′																			
*Ido1*				Fw: 5′-GACTTCGTGGATCCAGAC-3′									277 bp			48				(58)			
NM_02397.1				Rv: 5′-TCTAAGGAGGAGAGGAAG-3′																			
*Gapdh*				Fw: 5′-GCCAAGGTCATCCATGACAAC-3′									600 bp			55							
NM_017008.4				Rv: 5′-GTCCACCACCCTGTTGCTGTA-3′																			

* Meter/minute: The intensity is expressed in meter/minute and the duration in minutes (min). Both the healthy and the diabetic animals underwent this exercise training protocol.

The intensity of training from 10 m/min and 23 min (including 5 min of warm-up and 3 min of cool-down) in the first week increased to 26 m/min and 59 min in each session in the eighth week. To apply the principle of overload, an average of 6 min per week (about 1 min per day) was added to the duration of the exercise and 2 m/min to the intensity of the exercise. Each training session, after warming up (at a speed of 5 to 10 m/min), started at a speed of 10 m/min, and, every 3 min, 2 m/min was added to the speed of the treadmill to reach the desired weekly speed (**Table 1A**).

### 2.6 Measurement of blood Glc levels

To measure the BGL, fresh blood samples were collected from the rat tail vein, and Glc levels were then determined using a blood Glc meter (ACCU-CHEK^®^ Active, Roche Diagnostics, Mannheim, Germany) that uses test strips to assess a Glc oxidoreductase-mediated dye reaction, according to the manufacturer’s instructions. The BGL was measured on day 3 after STZ treatment and at the end of the eighth week of the training course. The measurements were performed in duplicate.

### 2.7 Tissue sample preparation

Tissue resection was performed at the end of the eighth week and 72 h after the last training session. This was after anesthesia with CO_2_ gas and blood sampling from the heart. Then, liver tissue was extracted and, after washing in physiological serum, placed in microtubes and transferred to −70°C.

### 2.8 Determination of malondialdehyde and KYN

Kynurenine ELISA Kit, ZellBio GmbH (cat. no. ZB-11203C-R9648, Germany), which applies quantitative assay of KYN based on the biotin double antibody sandwich technology, was used to determine KYN level in liver tissue lysate following the manufacturer’s instructions. In addition, the malondialdehyde (MDA) assay kit (cat. no. ZB-MDA-96A, Germany), which works based on the colorimetric (535-nm) method, was used for MDA assay in liver homogenate following the instructions. The determinations were performed in duplicate.

### 2.9 Gene expression analysis by real-time RT-PCR

Total RNA was isolated from liver samples using TRIzol Reagent (Invitrogen, USA), treated with DNase I, and quantified by NanoDrop (Thermo Fisher Scientific). RNA quality was determined by examining the 260/280 ratio >1.8. A total of 1 µg of RNA was then reverse-transcribed to cDNA using the RevertAid First Strand cDNA Synthesis kit (Thermo Fisher Scientific) according to the manufacturer’s protocol. Expression of *Ahr*, *Cyp1a1*, and *Ido1* was measured using specific primers purchased from SinaColon, Iran (**Table 1B**). CYP1A1 upregulation is a hallmark of AHR activation (62). Amplification was performed in duplicate in ABI Prism 7500 sequence detection system (Life Technologies real-time RT-PCR device). All data were analyzed with the ΔΔCt method, and the mRNA of glyceraldehyde-3-phosphate dehydrogenase (*Gapdh*) was used as the internal standard. The primer concentration was 10 μl. Amplification conditions were as follows: initial denaturation (95°C for 15 min for all genes); start of the cycle with denaturation (95°C for 30 s for *Gapdh*, and 95°C for 5 min for *Ahr*, *Cyp1a1*, and *Ido1*); annealing (55°C for 30 s for *Gapdh*, 48°C for 105 s for *Ido1*, 56°C for 90 s for *Cyp1a1*, and 60°C for 90 s for *Ahr*); and extension (60°C for 30 min for all genes). At the end of the amplification cycles, reactions were given a final extension step at 60°C–95°C. The relative percentage expressions of candidate genes in STZ-induced rats with diabetes were computed by considering the expression of *Gapdh* in Ct rats as 100%.

### 2.10 Protein level analysis by ELISA methods

ELISA kits from ZellBio GmbH (Germany) were utilized for determining AHR (cat. no. ZB-16349C-R; assay range, 0.313 to 10 ng/ml; sensitivity, 0.04 ng/mL), CYP1A1 (cat. no. ZB-11059C-R9648; assay range, 3 to 96 ng/ml; sensitivity, 0.35 ng/ml), and IDO1 (cat. no. ZB-10730C-R9648; assay range, 30 to 960 pg/ml; sensitivity, 3.5 pg/ml) protein levels in liver lysates. Enzyme-linked immunosorbent assays were performed using kits based on biotin double antibody sandwich technology. Reagents, samples, and standards were prepared following the manufacturer’s instructions. Final calculations were done using the standard curves provided by the kits for each protein. The determinations were performed in duplicate.

### 2.11 Statistical analysis

Data are presented as means ± standard deviations (SD) in the text and tables. In addition, for each variable, the percent change in each group compared with that in the Ct group was calculated (for example, percent change of the D group versus the Ct group was calculated as following formula: $\frac{mean\;of\;D\;group-mean\;of\;Ct\;group}{mean\;of\;Ct\;group}\times 100$ ), and its statistical significance was assessed using a one-sample t-test. Pearson and partial correlation coefficients were calculated to measure the relationship between AHR, CYP1A1, IDO1, KYN, and MDA, and protein levels.

A three-way multivariate analysis of variance (MANOVA) with all main and interaction terms was first performed to determine the effect of three factors (having diabetes, yes/no; exercise, yes/no; and nettle use, yes/no) on the latent variable. If the interaction term was not statistically significant, then it was removed from the model and the analysis rerun without the interaction term. Failure to remove an interaction term that was not statistically significant will lead to an incorrect conclusion.

In the next step, if the result of the multivariate analysis was statistically significant, then univariate three-way analysis of variance (ANOVA) were done to discover the effect of a significant factor on each indicator variable (AHR, CYP1A1, IDO1, KYN, and MDA for first latent variable; *Ahr*, *Cyp1a1*, and *Ido1* for second latent variable), which was followed by the Tukey’s *post hoc* test. Assumptions of three-way ANOVA were verified with the Shapiro–Wilk test, Levene’s test, Doornik–Hansen test, Box’s M-test, and evaluation of the homogeneity of covariance matrices. All statistical computations were done using Stata version 16 (StataCorp LLC, College Station, TX, 2019) and GraphPad Prism 8 (GraphPad Software Inc., La Jolla, California, USA). The results were considered significant with P ≤ 0.05.

## 3 Results

### 3.1 Phytochemical analysis of the nettle leaf extract

**Figure 1:**
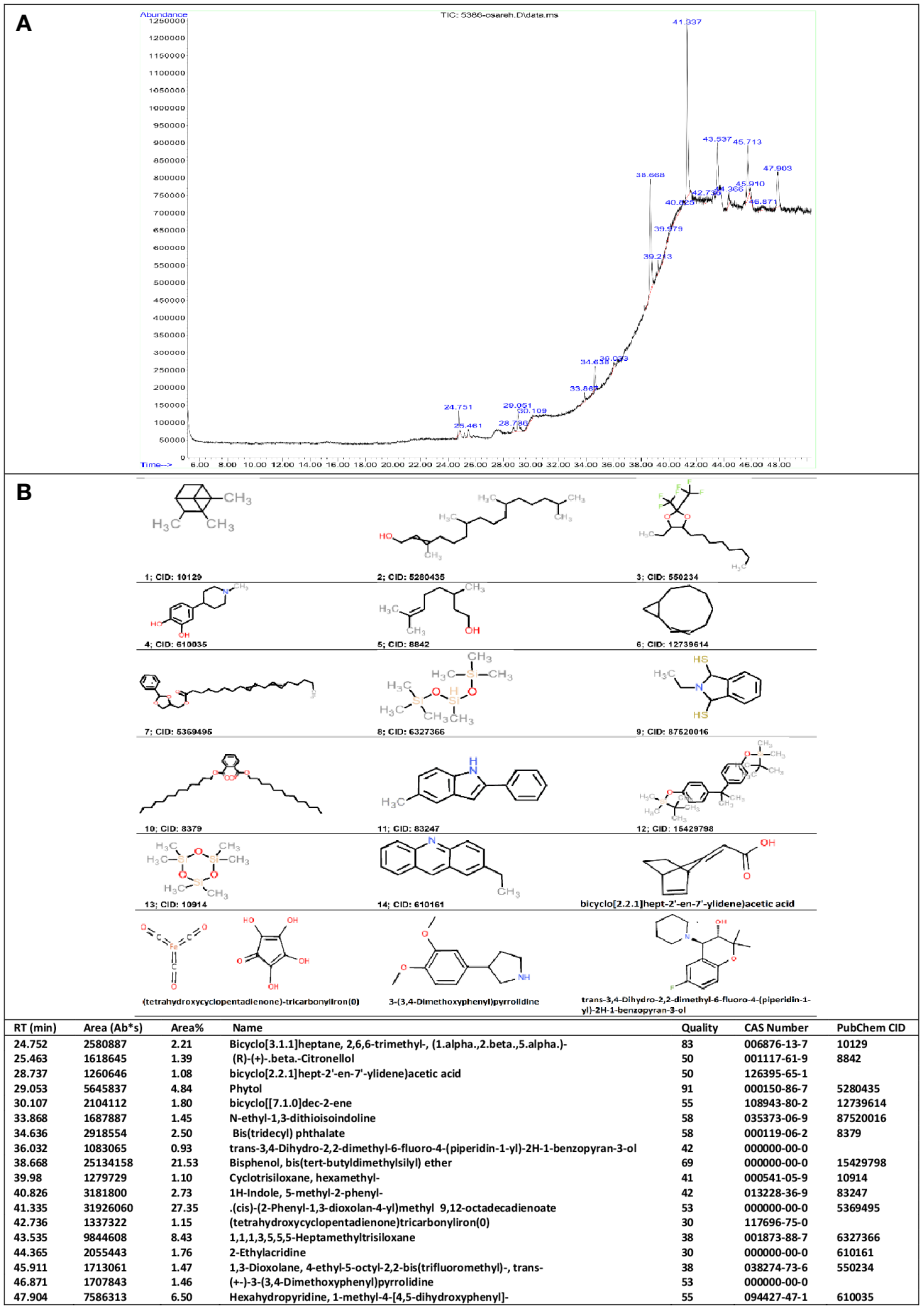
GC-MS results. Phytochemical constituents identified in the hydro-alcoholic extract of nettle leaf using gas chromatography-mass spectrometry **(A)**. Two-dimensional structure of the chemicals **(B)**.

As shown in **Figure 1A**, the GC-MS chromatogram of hydro-alcoholic extract of nettle leaf revealed peaks, which corresponded to 18 bioactive compounds identified by comparison with the known chemicals described in Wiley10th-NIST11b mass spectral database based on the peak retention time, peak area (%), peak height (%), and mass spectral fragmentation patterns. The following *in silico* target analysis includes chemicals (14 molecules) that have been found listed in the PubChem database with CID identifiers and those chemicals (four molecules) that we produced their SMILES notations using OPSIN (**Figure 1B**).

### 3.2 *In silico* target analysis and gene enrichment

A summary of the gastrointestinal (GI) absorption, water solubility, and bioavailability scores calculated by SwissADME, as well as their LD50s and toxicity classes predicted by ProTox-II, can be found in **Table 2** for the compounds with CID identifiers. In addition, SuperPred predicted that the chemicals had a different number of target genes (**Table 2**; **Supplementary File 1**). The greatest number of predicted target genes, 194, was associated with chemical 10129, and the smallest number, 80, was associated with chemical 8379, as shown in **Table 2**. Furthermore, ToppFun analyzed the predicted lists and identified the 50 most related diseases and abnormalities for each group of genes and ranked them (**Supplementary File 2**). The predictions include complications related to diabetes and associated diseases such as diabetic nephropathy, diabetic cardiomyopathies, metabolic diseases, non-alcoholic fatty liver disease, cardiovascular diseases, and hypertensive disease. In particular, hyperglycemia, which is directly related to diabetes, was predicted for 18 gene lists with varying rankings (**Table 2**; **Supplementary File 3**). The link between hyperglycemia and the target genes for chemicals 10129, 5280435, and 550234 came in third place among 50 diseases. On the other hand, hyperglycemia was not predicted to be associated with the target genes of chemicals 10914, 60161, and (tetrahydroxycyclopentadienone)-tricarbonyliron(0) chemicals. There is therefore a possibility that among the compounds analyzed, chemicals 10129, 5280435, and 550234 may play a more significant role in diabetes physiology. As far as GI absorption is concerned, these three chemicals are the same; however, 10129 has a greater water solubility and is the least toxic (LD50, 15,380 mg/kg) of the three, compared with 5280435 (LD50, 5,000 mg/kg) and 550234 (LD50, 800 mg/kg). It may therefore be worthwhile to investigate the effects of chemical 10129 on diabetes pathways through further analysis. Interestingly, IDO1 was listed among the target genes for chemicals 10129 and 550234 (**Supplementary File 1**). When all predicted target genes for all chemicals were combined, there were 462 unique genes, with hyperglycemia ranking fifth among the abnormalities assigned to them. The first four abnormalities were hypertension, pain, myocardial infarction, and cardiovascular diseases, which are conditions related to diabetes.

**Table 2:** Predicted characteristics for mass spectrometry identified phytochemical constituents of NLE.

PubChem CID/Name	GI absorption	Water solubility	Bioavailability score	Predicted LD50	Predicted toxicity class	Target genes (N)^1^	Rank of hyperglycemia^2^
10129	Low	Soluble	0.55	15,380 mg/kg	6	194	3
5280435	Low	Moderately soluble	0.55	5,000 mg/kg	5	137	3
550234	Low	Moderately soluble	0.55	800 mg/kg	4	186	3
610035	High	Soluble	0.55	348 mg/kg	4	132	8
Trans-3,4-Dihydro-2,2-dimethyl-6-fluoro-4-(piperidin-1-yl)-2H-1-benzopyran-3-ol	High	Soluble	0.55	1,500 mg/kg	4	153	8
(+-)-3-(3,4-Dimethoxyphenyl)pyrrolidine	High	Soluble	0.55	530 mg/kg	4	141	9
8842	High	Soluble	0.55	3,450 mg/kg	5	132	9
12739614	Low	Soluble	0.55	5,000 mg/kg	5	179	10
5369495	Low	Poorly soluble	0.55	3,520 mg/kg	5	94	10
6327366	High	Soluble	0.55	24,134 mg/kg	6	111	13
bicyclo[2.2.1]hept-2’-en-7’-ylidene)acetic acid	High	Soluble	0.85	5,000 mg/kg	5	77	13
87520016	High	Soluble	0.55	2,219 mg/kg	5	89	27
8379	Low	Insoluble	0.17	1,340 mg/kg	4	80	31
83247	High	Poorly soluble	0.55	100 mg/kg	3	128	34
15429798	Low	Poorly soluble	0.55	5,000 mg/kg	5	100	47
10914	High	Soluble	0.55	24,134 mg/kg	6	99	Not included
610161	High	Poorly soluble	0.55	940 mg/kg	4	109	Not included
(Tetrahydroxycyclopentadienone)-tricarbonyliron(0)	Low	Soluble	0.56	5,000 mg/kg	5	76	Not included
Combined	–	–	–	–	–	462	5

^1^Number of target genes of the chemical predicted by SuperPred.

^2^Hyperglycemia rank assigned by ToppFun to predicted target genes.

### 3.3 Exercise and NLE decreased blood Glc level

**Figure 2:**
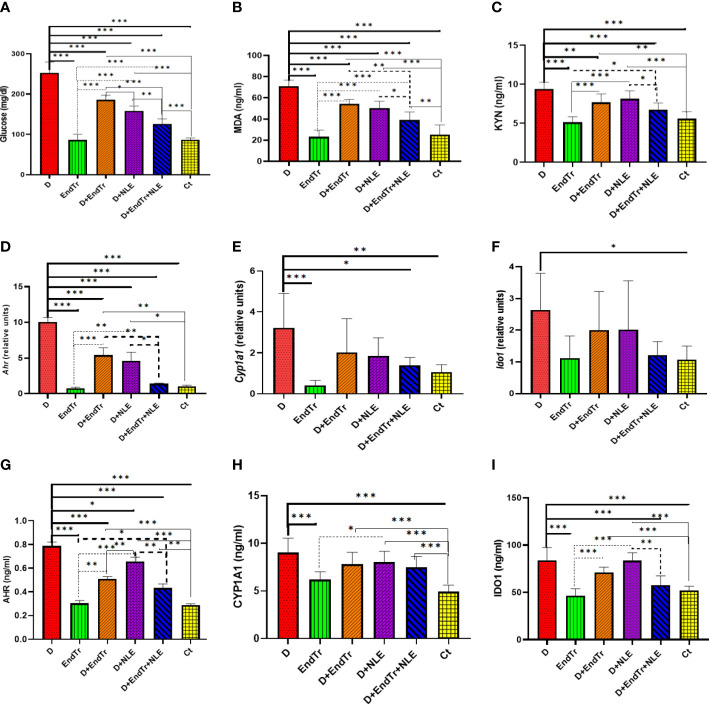
Levels of glucose in peripheral blood **(A)**; levels of MDA **(B)** and KYN **(C)**; expression of *Ahr* **(D)**, *Cyp1a1* **(E)**, and *Ido1* **(F)**; as well as levels of AHR **(G)**, CYP1A1 **(H)**, and IDO1 **(I)** proteins in the liver of rats in control and treated groups. Because there were different groups in the study, a total of 15 comparisons were conducted. Only statistically significant findings are presented in the figure, and different types of lines are assigned on the basis of the comparisons. Continuous tick line, significant comparisons of other groups with the D group; continuous thin line, the significant comparison of Ct group with other groups; dashed tick line, significant differences of the D + EndTr + NLE with others; and dashed thin lines, significant comparisons of other with the EndTr group. Abbreviations: D (group with STZ-induced diabetes), EndTr (group with endurance training), D + EndTr (rats with STZ-induced diabetes treated with EndTr), D + NLE (rats with STZ-induced diabetes treated with NLE), D + EndTr + NLE (rats with STZ-induced diabetes treated NLE and EndTr), and Ct (control group). *: P<0.05, **: P<0.01, ***: P<0.001.

As shown in **Figure 2A**, Glc levels were determined in the blood of rats in different study groups. The Glc level in the D group was 252.5 ± 27.10 mg/dl, which was significantly higher than the Glc level in the Ct group (86.75 ± 4.30 mg/dl). Therefore, diabetes was successfully induced in rats using STZ. In **Figure 2A**, it can be seen that the BGLs of the study groups differed significantly. Both the D + EndTr and D + NLE groups showed a significant decrease in BGL (P< 0.001). Nevertheless, the combination of EndTr and NLE in the D + EndTr + NLE group reduced BGL more than their individual applications. As a result, BGL was reduced to 126.25 ± 5.40 mg/dl, which was significantly lower than in D (P< 0.001), D + EndTr (P< 0.001), and D + NLE (P = 0.002) groups. In addition, the difference in BGL between the D + NLE and D + EndTr groups was significant (P = 0.011). However, although BGL was significantly lower in the D + EndTr, D + NLE, and D + EndTr + NLE groups than that in the D group, it was still significantly higher than that in the Ct group. Comparison of EndTr and Ct groups, indicated that EndTr had no effect on BGL of healthy rats.

### 3.4 Exercise and NLE decreased elevated levels of MDA and KYN

According to **Figure 2B**, MDA, a biomarker of oxidative stress, was significantly higher in the liver tissues of the D group in comparison with controls (P< 0.001). However, its level was significantly (P< 0.001) reduced by EndTr (in the D + EndTr group) and NLE (in the D + NLE group). In the D + EndTr + NLE group, MDA levels were significantly lower than in the D (P< 0.001), D + EndTr (P = 0.001), and D + NLE (P< 0.032) groups. It should be noted that there was no significant difference between the MDA levels in the D + NLE and D + EndTr groups, and compared with the Ct group, EndTr did not result in an increase in MDA levels in healthy rats’ livers.

In the D group (**Figure 2C**), KYN levels significantly elevated as compared with controls (P = 0.001). However, a significant reduction in KYN levels was observed in the D + EndTr group (P = 0.006). Despite the lower KYN level recorded in the D + NLE (8.15 ± 0.32 ng/ml) and D + EndTr (7.67 ± 0.32 ng/ml) groups in comparison with the D group (9.41 ± 0.32 ng/ml), the differences were not statistically significant. However, the combined application of EndTr and NLE (in the D + EndTr + NLE group) resulted in significantly (P = 0.046) lower levels of KYN (6.73 ± 0.87 ng/ml) in comparison with using NLE alone. In addition, because there was no significant difference between the EndTr and control groups, it can be concluded that exercise did not affect the levels of KYN in the liver of healthy rats.

### 3.5 Exercise and NLE decreased elevated expression of *Ahr*, *Cyp1a1*, and *Ido1*

The results of real-time RT-PCR tests revealed that *Ahr*, *Cyp1a1*, and *Ido1* expression levels significantly increased in the livers of rats in the D group (**Figure 2D**). It is notable that, although EndTr (P<0.001) and NLE (P< 0.001) have a substantial effect on the downregulation of *Ahr* expression, there were still significant differences between controls and the treated D + EndTr and D + NLE groups (**Figure 2D**). Despite this, their combination reduced *Ahr* expression to a level near that of controls (**Figure 2D**). Although EndTr and NLE reduced *Cyp1a1* expression in the liver, there were no significant differences between the D group and the D + EndTr and D + NLE groups (**Figure 2E**). The combination of EndTr and NLE, however, resulted in a significant decrease in *Cyp1a1* expression (P = 0.018). The expression of the *Ido1* gene was also downregulated in the D + EndTr, D + NLE, and D + EndTr + NLE groups; however, the differences were not statistically significant (**Figure 2F**). In addition, exercise did not significantly alter the expression of these genes in the liver of healthy rats.

### 3.6 Exercise and NLE affect levels of *AHR*, *CYP1A1*, and *IDO1* proteins

AHR, CYP1A1, and IDO1 levels significantly increased in the liver of the D group as determined by ELISA tests (**Figure 2G**). There was a significant effect of EndTr (P< 0.001) and NLE (P = 0.021) on the downregulation of AHR protein expression (**Figure 2G**). In addition, in the D + EndTr + NLE group, AHR levels significantly reduced (P = 0.001), although there were still significant differences between the control and treated groups (D + EndTr, D + NLE, and D + EndTr + NLE). Neither EndTr nor NLE or their combination significantly reduced the increased level of CYP1A1 (**Figure 2I**). An analysis of the liver CYP1A1 levels of healthy rats in the EndTr and Ct groups showed that EndTr did not significantly elevate liver CYP1A1 levels. In terms of IDO1, its level was significantly lower in the D + EndTr + NLE group compared with the D and D + NLE groups, as shown in **Figure 2I** (P< 0.001).

### 3.7 Analysis of synergistic effects of EndTr and NLE

In **Table 3**, descriptive statistics and percent change of assessed variables in the liver are presented for each of the six groups. On the basis of this table, AHR, CYP1A1, KYN, and MDA showed the greatest percent change in the D group, whereas IDO1 showed the greatest percent change in both the D and D + NLE groups. Combining EndTr and NLE may reduce elevated levels of AHR, CYP1A1, KYN, IDO1, and MDA in diabetic conditions in a synergistic manner, as outlined in the table. There was no significant violation of the assumption of multivariate normality [χ2 (10) = 16.23, P = 0.093]. Furthermore, Box’s M-test showed that the observed covariance matrices of the dependent variables were equal across groups (P = 0.064).

**Table 3:** Descriptive statistics and percent change of variables in the six groups.

Group	Control	AHR	IDO1	CYP1A1	KYN	MDA
EndTr	Mean (SD)	0.31 (0.07)	46.50 (7.36)	6.20 (0.81)	5.14 (0.68)	23.13 (6.33)
	% Change	7.43	-9.69	28.99	-6.42	3.28
	P-value^*^	0.463	0.218	0.025	0.373	0.844
D	Mean (SD)	0.79 (0.09)	83.81 (13.50)	9.05 (1.50)	9.41 (0.85)	71.06 (5.63)
	% Change	176.71	60.68	88.41	72.61	228.68
	P-value	<0.001	<0.001	0.001	0.001	0.003
D + EndTr	Mean (SD)	0.51 (0.06)	71.00 (5.62)	7.82 (1.23)	7.67 (1.10)	54.19 (4.44)
	% Change	78.53	36.77	59.39	40.86	153.52
	P-value	<0.001	<0.001	<0.001	0.015	0.009
D + NLE	Mean (SD)	0.66 (0.10)	83.59 (8.20)	8.07 (1.08)	8.15 (1.01)	50.19 (6.52)
	% Change	129.18	60.67	69.19	49.79	125.45
	P-value	<0.001	<0.001	0.003	0.007	0.004
D + EndTr + NLE	Mean (SD)	0.43 (0.09)	57.69 (9.83)	7.50 (1.13)	6.73 (0.87)	39.06 (7.56)
	%Change	53.76	11.37	52.77	23.47	78.25
	P-value	0.010	0.218	<0.001	0.062	0.021
Ct	Mean (SD)	0.29 (0.03)	52.22 (4.28)	4.92 (0.70)	5.61 (0.86)	25.06 (9.34)

D (group with STZ-induced diabetes), EndTr (group with endurance training), D + EndTr (STZ-induced diabetic rats with EndTr), D + NLE (STZ-induced diabetic rats treated with nettle leaves extract), D + EndTr + NLE (STZ-induced diabetic rats treated with NLE and EndTr), and Ct (control group). % Change: The percent change average of each group related to the control grou. * Based on one sample T-test (percent change = 0).

On the basis of the results of **Table 4**, a significant effect of exercise × NLE × diabetes (Wilks’ λ = 0.35, partial eta square = 0.41, P< 0.001) was observed on the latent variables (AHR, CYP1A1, KYN, IDO1, and MDA simultaneously). This impact was considered in the individual univariate analysis, which indicated significant effects on AHR [F(2,42) = 16.46, P< 0.001], CYP1A1 [F(2,42) = 5.50, P = 0.008], KYN [F(2,42) = 4.53, P = 0.010], IDO1 [F(2,42) = 5.59, P = 0.007], and MDA [F(2,42) = 4.89, P = 0.012]. Although according to this table, the main effects of exercise, NLE, and diabetes, as well as the interaction effect of exercise and diabetes, were significant, but in cases where the three-way interaction effect is significant, these main and two-way effects should not be interpreted. The significance of three-way interaction of exercise, NLE, and diabetes means that the interaction among the two factors (exercise × NLE) is different across the levels of the third factor (diabetes) for AHR, CYP1A1, KYN, IDO1, and MDA. Similar findings were obtained for gene expression of *Ahr*, *Ido1*, and *Cyp1a1* (**Table 5**).

**Table 4:** Three-way MANOVA and ANOVA results for protein expression of AHR, IDO1, and CYP1A1 and level of KYN and MDA.

Factor	Three-way MANOVA Results			Three-way ANOVA results			
	Wilks’ lambda	η^*^	P-value	Dependent variable	F (1, 42) ^#^	η	P-value
Exercise	0.37	0.63	<0.001	AHR	24.11	0.66	<0.001
				IDO1	20.64	0.46	<0.001
				CYP1A1	0.32	0.11	0.026
				KYN	13.88	0.37	<0.001
				MDA	14.45	0.44	<0.001
Diabetes	0.06	0.94	<0.001	AHR	165.41	0.46	<0.001
				IDO1	77.45	0.37	<0.001
				CYP1A1	54.16	0.22	0.001
				KYN	99.83	0.47	<0.001
				MDA	253.45	0.71	<0.001
NLE	0.30	0.70	<0.001	AHR	14.52	0.25	<0.001
				IDO1	4.51	0.10	0.040
				CYP1A1	2.75	0.06	0.104
				KYN	12.11	0.22	0.001
				MDA	55.31	0.56	<0.001
Exercise × Diabetes	0.45	0.56	<0.001	AHR	31.88	0.43	<0.001
				IDO1	6.11	0.12	0.018
				CYP1A1	10.37	0.19	0.002
				KYN	4.11	0.09	0.049
				MDA	8.28	0.16	0.006
Exercise × NLE × Diabetes	0.35	0.41	<0.001	AHR	16.46	0.44	<0.001
				IDO1	5.59	0.21	0.007
				CYP1A1	5.50	0.21	0.008
				KYN	4.53	0.20	0.010
				MDA	4.89	0.19	0.012

* Partial eta squared. # For three-way interaction (exercise × diabetes × NLE) is (2, 42). NLE, Nettle leaf extract.

**Table 5:** Three-way MANOVA and ANOVA results for gene expression of *Ahr*, *Ido1*, and *Cyp1a1*.

Factor	Three-way MANOVA results			Three-way ANOVA results			
	Wilks’ Lambda	η^*^	P-value	Dependent variable	F (1, 42)	η	P-value
Exercise	0.56	0.44	<0.001	*Ahr*	19.16	0.30	<0.001
				*Ido1*	2.59	0.06	0.115
				*Cyp1a1*	6.52	0.13	0.014
Diabetes	0.24	0.76	<0.001	*Ahr*	80.40	0.65	<0.001
				*Ido1*	11.96	0.21	0.001
				*Cyp1a1*	26.02	0.37	<0.001
NLE	0.41	0.59	<0.001	*Ahr*	38.24	0.47	<0.001
				*Ido1*	3.97	0.08	0.053
				*Cyp1a1*	7.26	0.14	0.010
Exercise × Diabetes	0.81	0.19	0.033	*Ahr*	14.27	0.49	<0.001
				*Ido1*	3.87	0.21	0.015
				*Cyp1a1*	7.28	0.33	<0.001
Exercise × Diabetes × NLE	0.14	0.49	<0.001	*Ahr*	26.38	0.76	<0.001
				*Ido1*	3.23	0.28	0.015
				*Cyp1a1*	6.60	0.44	<0.001

* Partial eta squared. NLE, Nettle leaf extract.

It should be noted that, in addition to **Figure 2**, which shows the significant comparison of all groups, to clarify the comparisons, two groups that had a significant difference in only one factor were given in **Figure 1s** of Supplemental section.

**Table 6A** indicates that there was a highly positive significant correlation between the studied factors based on the ***r*** coefficient. Moreover, in the bivariate analysis, there was a moderately positive correlation between factors (based on ***r_p_*** coefficient), but it was significant only for some of them (**Table 6A**). **Figure 2** presents the results of the Tukey’s *post hoc* test and pairwise comparisons of groups. In addition, **Table 6B** presents the parameter estimates of variables in the six study groups. The control group was considered as a reference group in this analysis, and the other groups were compared with it. Resulting from the regression model, diabetes elevated all assessed variables in the liver, and the combination of EndTr and NLE significantly reduced these factors. Comparing the effects of EndTr and NLE (considering the **β** coefficient) suggests that EndTr affects all variables except MDA more strongly than NLE (**Table 6B**).

**Table 6:** Relationships between variables and parameter estimates.

(A) The relationships between variables in liver tissue by Pearson and partial correlation										
Variable	Correlation	AHR	IDO1		CYP1A1		KYN		MDA	
			Coef	P-value	Coef	P-value	Coef	P-value	Coef	P-value
AHR	r	1	0.83^*^	<0.001	0.71	<0.001	0.79	<0.001	0.82	<0.001
	r_p_		0.46	0.001	0.30	0.043	0.21	0.171	0.31	0.040
IDO1	r		1		0.56	0.001	0.75	<0.001	0.77	<0.001
	r_p_				-0.18	0.249	0.26	0.083	0.23	0.123
CYP1A1	r				1		0.66	<0.001	0.68	<.001
	r_p_						0.20	0.191	0.24	0.117
KYN	r						1		0.75	<0.001
	r_p_								0.19	0.222
MDA	r								1	
	r_p_									
(B) Parameter estimates of variables in the six groups										
Group	Control	AHR		IDO1	CYP1A1		KYN		MDA	
D	β	0.50		31.59	4.125		3.80		46.00	
	P-value	<0.001		<0.001	<0.001		<0.001		<0.001	
EndTr	β	0.017		−5.72	1.28		-0.47		-1.94	
	P-value	0.451		0.194	0.026		0.309		0.572	
D + EndTr	β	0.223		18.78	2.90		2.06		29.13	
	P-value	<0.001		<0.001	<0.001		<0.001		<0.001	
D + NLE	β	0.369		31.38	3.15		2.54		25.13	
	P-value	<0.001		<0.001	<0.001		<0.001		<0.001	
D + EndTr + NLE	β	0.146		5.47	2.58		1.12		14.00	
	P-value	0.001		0.214	<0.001		0.017		<0.001	

r, Pearson correlation; r_p_, partial correlation; Coef, coefficient; β, parameter estimates; Ct, reference category.

## 4 Discussion

The effects of NLE and EndTr on the IDO1-KYN-AHR pathway in the liver of diabetic patients are not well understood at present. On the basis of the results presented here, models with STZ-induced diabetes exhibited significantly higher levels of MDA and KYN in liver tissue, consistent with previous observations (63). Known as a biomarker of oxidative stress, MDA is an extremely toxic byproduct formed when free radicals react with phospholipids and cause to lipid peroxidation (64). In addition, it has been shown that KYN levels are related to MDA production, and exogenous KYN treatment induces the oxidative pathway and results in the peroxidation of lipids (65).

In this study, we showed that NLE reduced the elevated levels of BGL and liver MDA in rats with STZ-induced diabetes, but not the liver KYN level (**Figures 2A–C**). Moreover, NLE had a significantly greater effect on lowering BGL than EndTr. Studies have demonstrated that NLE mediated cytoprotection by significantly increasing anti-oxidant activity and reducing lactate dehydrogenase activity and MDA formation in Swiss albino mice livers (66). In relation to diabetes, NLE has been reported to have insulin secretagogue, PPARgamma antagonistic, and alpha-glucosidase inhibiting properties and may help improve glycemic control (67). A systematic review and meta-analysis published by Ziaei et al. suggests that nettle supplementation may be effective in controlling T2D (68).

The current study found that EndTr significantly reduced elevated BGLs and liver MDA in the models, which is consistent with previous studies regarding the effects of exercise on patients with T2D (35). Nevertheless, their levels in the models were still significantly higher than in the controls after training courses, and the same results were observed for NLE treatments as well. It has been suggested by Taysi et al. that EndTr attenuates oxidative stress in the liver, probably through preventing decreases in glutathione peroxidase and superoxide scavenger activity (69).

It is important to note here that STZ itself induces oxidative damage and cytotoxicity in hepatocytes (70–72). Nonetheless, these observations do not imply that STZ-induced perturbations in Glc metabolism and hyperglycemia do not contribute to the observed oxidative stresses and cytotoxic results. In this regard, Chen et al. examined liver samples from rats with diabetes induced by STZ and found that hepatic metabolic pathways for Glc, choline-betaine-methionine, and amino acids were disturbed during diabetes progression. A more important finding was that the levels of choline, betaine, and methionine in the livers of the rats decreased, which suggests that hyperglycemia undermines the protective function of the liver in response to liver injury (73). Therefore, there may be overlaps between STZ-induced oxidative stress and hyperglycemia-induced oxidative stress in hepatocytes.

As KYN emerges as a key player in diseases, KP is being considered as a possible therapeutic target for diabetes (24, 39). The concentrations of KP metabolites differ significantly between patients with T1D and T2D, including significantly elevated levels of anthranilic acid (derived from KYN), which has not been observed among patients with T2D, and higher levels of kynurenic acid and xanthurenic acid compared with patients with T2D (4). Anthranilic acid has been shown to cause an elevation of TRP and dysregulation of the autoimmune process in T1D as a result of the downregulation of IDO (25, 74). In addition, increased levels of kynurenic acid were associated with the development of T1D in the non-obese mouse with diabetes model (75). Furthermore, impaired TRP catabolism and elevated levels of related metabolites such as KYN have also been reported in children with T1D, which indicates that there remains a systemic inflammatory response (15). Despite this, some studies have suggested that increasing KP metabolites in the liver may protect against type 1 diabetes and promoting KYN and kynurenic acid production might be possible to achieve these anti-diabetic benefits (4, 76). Therefore, maintaining homeostasis of this pathway is crucial to the control of diabetes.

The present study found that EndTr significantly decreased the levels of KYN in the liver of rats with diabetes in the D + EndTr group, but they were still significantly higher than those in controls. However, in the D + EndTr + NLE group, liver KYN levels were low enough to not differ significantly from those in control rats. Exercise has been shown to affect KP in various cancers. Following exercise, serum KYN levels and the KYN/TRP ratio significantly reduced in patients with breast cancer (77). In addition, resistance exercises may enhance KP and decrease KYN/TRP levels in patients with pancreatic cancer, modulating their immune system (78).

As rate-limiting enzymes, IDO1, IDO2, and TDO catalyze the conversion of TRP into KYN and are upregulated in patients with T2D (25, 79). As far as T1D is concerned, current studies on immunotherapies describe the use of dendritic cells and IDO to control the progression of the immune responses that trigger the disease (80). T1D animal models with impaired expression and activity of IDO are unable to develop immune tolerance to autoantigens (16). The IDO1 function in sera and peripheral blood mononuclear cells of children with T1D is also significantly impaired (17). Moreover, the absence or remarkable decrease of expression of the IDO1 gene in pancreatic islets prior to the onset of T1D could contribute to the disease (19). It has also been shown that upregulation of IDO and elevated levels of KP metabolite QA may contribute to the development of diabetic retinopathy in individuals with T1D (18). There is some promising evidence that restoring IDO function can restore normoglycemia in T1D, and IDO-involved pathways, such as the TLR9-IDO axis, may be valuable targets in the prevention and treatment of the disease (17, 20).

KYN is known to be an AHR agonist, and AHR is crucial for liver function (81). There is also substantial evidence that AHR may contribute to the development of T1D due to its widespread expression in immune cells and its ability to modulate autoimmune responses (22). The complex formation of AHR together with many transcriptional factors controls the expression of critical genes relevant to the development of T1D (82). According to an interesting study, combined with EndTr, resveratrol, an AHR inhibitor, can significantly reduce the inflammatory factor TNF-alpha in rats with T2D and reduce their fasting Glc levels (83). AHR, IDO1, and CYP1A1 levels in the liver of the EndTr group did not differ significantly from those of the control group. Therefore, EndTr did not disrupt the IDO1-KYN-AHR pathway in controls. However, both EndTr and NLE significantly reduced AHR levels in the liver of models (D + EndTr, D + NLE, and D + EndTr + NLE groups), with EndTr being significantly more effective than NLE. The reducing effect of the combined EndTr and NLE was significantly stronger than NLE.

Many plant compounds have been shown to affect the AHR pathway, and NLE contains substances that may be antagonistic or agonistic to the pathway (84–86). In addition, our *in silico* analysis revealed that 18 chemicals identified in the NLE can interact with a wide range of different target genes (**Supplementary File 1**). These genes are predicted to play a role in a wide range of abnormal conditions, including diabetes complications and particularly hyperglycemia (**Supplementary Files 2**, **3**). Chemical 10129 may, based on compared characteristics, have some distinct effects on diabetes-related pathways, as described in the results section.

Several studies have recently examined the synergistic effects of exercise and herbal medicines on mitigating the symptoms of diabetes (87). In one study, Ghalavand et al. found that aerobic interval training together with nettle supplementation was effective in controlling blood sugar and blood pressure in patients with T2D, and their combination proved more effective than either of them alone (88). Despite the limited number of studies related to T1D, this approach has also been recommended for the management of its complications (89, 90). In the current study, we showed that, in terms of lowering BGL and liver MDA levels, the combined administration of NLE and EndTr was significantly more effective than their separate administration. Nevertheless, when it comes to liver KYN, the combination had a greater reduction effect than NLE alone. This suggests that exercise is more effective than NLE alone in reducing liver KYN levels. However, there were no significant differences in MDA levels between the D + NLE and D + EndTr groups. Delavar et al. concluded that aerobic interval training and nettle supplementation both have a beneficial effect on blood Glc control and inflammation reduction, and the combination of these two methods appears to be more effective in improving blood Glc control in those with T2D (91).

Because hyperglycemia does not occur immediately after STZ treatment, diabetes induction in the model could be divided into acute (before hyperglycemia) and subacute (after hyperglycemia) phases. The liver may undergo changes such as lipid peroxidation, mitochondrial swelling, peroxisome proliferation, and inhibition of hepatocyte proliferation before serum Glc levels increase (70). There is, however, a correlation between the changes in hepatocytic changes in the acute and subacute phases, and the signaling pathways that are directly stimulated by hyperglycemia appear to have a crucial role in the production of reactive oxygen species, oxidative stress, and cell death (92). One limitation of the present study is the inability to compare changes during the acute and subacute phases of diabetes induction so as to distinguish the effects of EndTr and NLE on STZ-induced changes and those caused by hyperglycemia. Furthermore, other analyses such as Glc tolerance testing, insulin tolerance testing, plasma insulin assay, histological analysis to examine liver morphology, and assessing liver-specific biomarkers such as those related to fat deposition, fibrosis, inflammation, and oxidative stress could provide a more comprehensive understanding of this study. Moreover, our observations do not appear to be related to the direct effect of NLE and EndTr on the KYN level and the IDO1-KYN-AHR pathway, but they may influence some other upstream factors, which may result in KYN levels decreasing and the pathway restoring in the liver by changing these factors. The molecular implications of the observed effects require further investigation into these topics, and the next steps in the study may include identifying the interactions between genes involved in lipid metabolism (SREBP1c and FASN), inflammation (F4/80 receptor, IFNγ, and TNFα), fibrosis (TGFβ andαSMA), and oxidative stress (NOX1 and NOX4) and IDO1-KYN-AHR axis in diabetic liver treated with NLE. In addition, the future clinical applications of the current study in T1D patients could include using NLE and EndTr for analyzing diabetes complications and liver function. It is also possible to develop more effective treatment programs by conducting human studies and clinical trials, as well as comparing the results between controls, patients with T1D and patients with T2D.

## 5 Conclusion

This study concluded that EndTr and NLE may have synergistic effects in reestablishing the homeostasis of the IDO1-KYN-AHR pathway in the liver of rats with diabetes. Although NLE had a significantly greater effect on lowering BGL than EndTr, training significantly reduced assessed factors in the liver except MDA more strongly than NLE. However, more detailed molecular studies especially using suppressors and activators of the pathway are essential for providing in-depth knowledge.

## Data availability statement

The raw data supporting the conclusions of this article will be made available by the authors, without undue reservation.

## Ethics statement

The animal study was reviewed and approved by IR.SEMUMS.REC.1399.291. All experimental protocols were approved by the Regional Research Ethics Committee of Semnan University of Medical Sciences and Health Services (IR.SEMUMS.REC.1399.291) (**Supplementary File 4**). The study was conducted following the Guide for the Care and Use of Laboratory Animals published by the US National Institutes of Health.

## Author contributions

YA, SD, MG, and RH have made substantial contributions to all of the following: (1) the conception and design of the study, acquisition of data, analysis, and interpretation of data, (2) drafting the article, revising it and (3) final approval of the version to be submitted.
